# Modulation of tumorigenesis by the pro-inflammatory microRNA miR-301a in mouse models of lung cancer and colorectal cancer

**DOI:** 10.1038/celldisc.2016.22

**Published:** 2016-06-21

**Authors:** Xiaodong Ma, Fang Yan, Qipan Deng, Fenge Li, Zhongxin Lu, Mofang Liu, Lisheng Wang, Daniel J Conklin, James McCracken, Sanjay Srivastava, Aruni Bhatnagar, Yong Li

**Affiliations:** 1 Department of Cancer Biology, Lerner Research Institute, Cleveland Clinic, Cleveland, OH, USA; 2 Department of Biochemistry and Molecular Biology, School of Medicine, University of Louisville, Louisville, KY, USA; 3 Diabetes and Obesity Center, Department of Medicine, University of Louisville, Louisville, KY, USA; 4 Institute of Pharmaceutical Research, South China Normal University, Guangzhou, China; 5 Department of Histology and Embryology Southern Medical University, Guangzhou, China; 6 Department of Medical Laboratory and Central Laboratory, The Central Hospital of Wuhan, Wuhan, China; 7 Institute of Biochemistry and Cell Biology, Shanghai Institutes of Biological Sciences, Chinese Academy of Sciences, Shanghai, China; 8 Department of Gastroenterology, Shenzhen Municipal People’s Hospital, Jinan University of Medical Sciences, Shenzhen, China

**Correction to:** *Cell Discovery* (2015) **1**, 15005; doi:10.1038/celldisc.2015.5; published online 19 May 2015

During web production, there was an error in Supplementary information: Supplementary Tables S1 and S2 were omitted. The files are now installed in the online version of the paper.

We apologize for any inconvenience that may have been caused by this error.

